# Editorial: Advancing science in support of sustainable bio-innovation: 16th ISBR Symposium

**DOI:** 10.3389/fbioe.2025.1597232

**Published:** 2025-04-10

**Authors:** Karen Hokanson, Detlef Bartsch, Monica Garcia-Alonso, Aparna Islam, Andrew Roberts, Jörg Romeis

**Affiliations:** ^1^ Agriculture & Food Systems Institute, Washington, DC, United States; ^2^ Federal Office of Consumer Protection and Food Safety (BVL), Berlin, Germany; ^3^ Estel Consult Ltd, Berkshire, United Kingdom; ^4^ BRAC University, Dhaka, Bangladesh; ^5^ Research Division Agroecology and Environment, Agroscope, Zürich, Switzerland

**Keywords:** biosafety, ISBR symposium, biotechnology regulation, GMO risk assessment, bio-innovation

**FIGURE 1:**
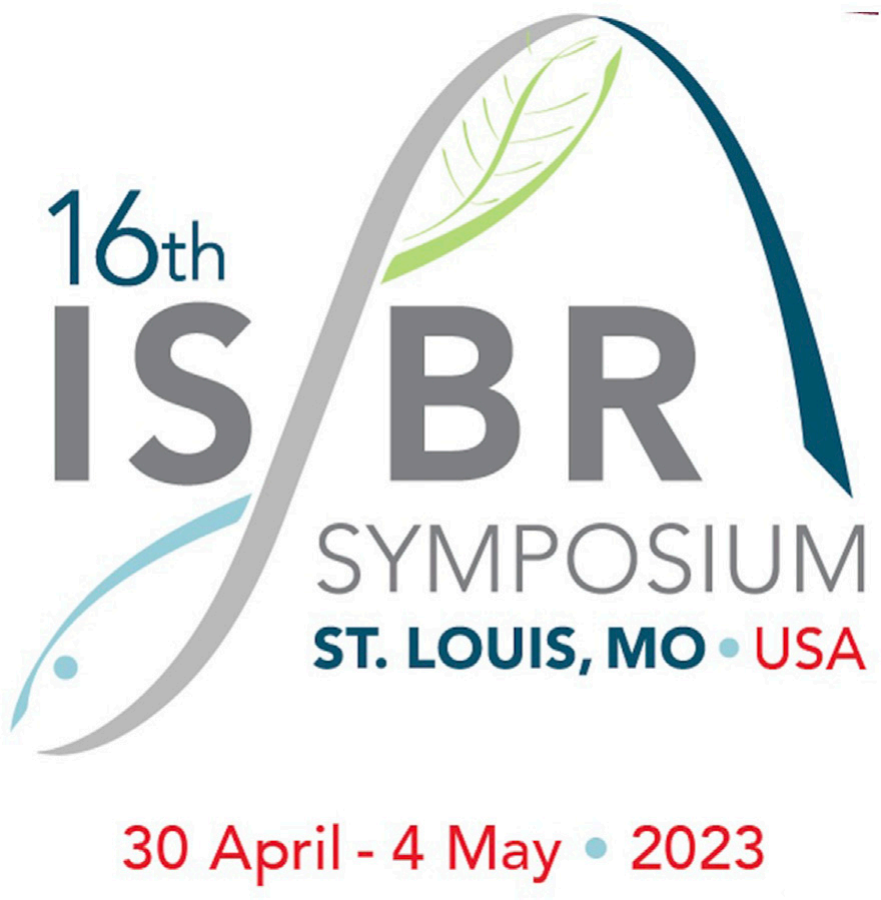
The 16th ISBR Symposium was held in May 2023 in St. Louis, Missouri, United States.

The ISBR Symposium is an international meeting organized biennially by the International Society for Biosafety Research (ISBR) since 1990 at various locations throughout the world (www.isbr.info). Aim of the symposia is to provide a unique opportunity for public and private sector research scientists, regulators, technology developers, non-government organizations and others to share their experience and expertise and to discuss biosafety related to the sustainable application of biotechnology. As with past symposia, ISBR hosted a Research Topic in Frontiers in Bioengineering and Biotechnology: section Biosafety and Biosecurity, open to the presenters at the most recent 16th ISBR Symposium held in May 2023 in St. Louis, Missouri, USA (Figure 1).

Around the central theme of ‘Advancing Science in Support of Sustainable Bio-innovation’, the 16th ISBR Symposium program included a series of presentations in four topical plenary sessions: 1) Ensuring social license for bio-innovation; 2) Risk analysis for persistent engineered genetic traits; 3) Fit-for-purpose governance frameworks for sustainable bio-innovation; and 4) Sustainable biotechnologies for a changing world. In addition to these plenary sessions, the symposium included over 20 organized sessions and workshops offered in parallel on a range of topics, and numerous Pecha Kucha and traditional poster presentations. Out of the diverse presentations, the society has assembled a Research Topic of 17 peer-reviewed publications representative of the different topics presented and discussed at the ISBR symposium.

As the ISBR Symposia aspire to delve broadly into considerations of biosafety policy as well as research, included in the Research Topic from the 16th ISBR Symposium are reviews discussing innovative approaches to the regulation of biotechnology. Two of these articles, by Storer et al. on modernizing and harmonizing regulatory data requirements for genetically modified crops and by Koch et al. on improving regulatory efficiency for biotechnology products, cover an important and recurring theme for the ISBR about using the growing years of experience with regulation of GM crops to make the regulatory process and decision-making more efficient and effective. Pence et al. also wrote about applying knowledge and experience from potato to update genetic stability data requirements in the risk assessment for cases of vegetatively propagated biotech crops.

Another review article by Nakai et al. is about the concept and scientific justification for data transportability for confined field trials conducted to support the risk assessment of GM plants. Data transportability, as a concept, considers the use of laboratory and field data generated on GM plants in one country to support the risk assessment of GM plants in another country, minimizing unnecessary duplication of regulatory studies and increasing efficiency in regulatory decision-making. The concept is being adopted in more and more countries and regions as experience with GM crops increases. Candia et al. also touch upon data transportability as a way to improve efficiency in an article describing the biosafety system in Paraguay and how the system might be improved taking experiences with its implementation into consideration.

Several articles also highlighted technical and policy issues related to some of the newer gene-editing techniques which require a different approach to regulation from that established for what is now thought of as ‘traditional’ genetically engineered organisms with inserted genes. Among these articles is a second paper with insights from Paraguay, by Rios et al., on the regulatory landscape for new breeding techniques (NBT) such as gene-editing. Goralogia et al. discuss rare but diverse off-target and somatic mutations found in field and greenhouse grown trees expressing CRISPR/Cas9. CRISPR (Clustered Regularly Interspaced Short Palindromic Repeats) is one of the most common tools used for gene-editing. One review article by Tripathi et al. discusses the use of CRISPR/Cas-based gene editing for improvement in one specific crop, bananas. Another article related to gene-editing, by McFadden et al., presents the results of a study to determine U.S. public opinion about the safety of gene editing in the agriculture and medical fields and the amount of evidence needed to improve opinions. A related opinion article by Lunshof discusses whether ‘social license’, as it has been used in the past, is obtainable in the case of novel bioinnovations that will be deployed in a shared environment, and suggests there might be more appropriate scenarios for involving all stakeholders in responsible deployment of novel bioinnovation.

Other articles presented more technical information for biosafety risk assessment. Avisar et al. share a risk assessment evaluation perspective in a research article on GM eucalyptus expressing pesticidal proteins from *Bacillus thuringiensis* for insect resistance. Mukani et al. present the results of a study on the nutritional composition analysis of GM potatoes developed in Kenya with resistance to late blight resistance, nutritional composition being a data set and analysis typically required for food safety assessment of GM crops. Two other studies, both from Bangladesh, present the analysis of baseline information management of fertilizer practices in potato (Nahiyan et al.) and crop weed management (Islam et al.), to inform the development and risk assessment of GM crops in this country.

Ahmed et al. share a perspective on understanding public perspectives on GM brinjal and the adoption of modern biotechnology in Bangladesh. Two more perspective articles in the Research Topic deal specifically with biotechnology and biosafety education. One from Diaz et al. is about Building bio-innovation systems through advanced biotechnology education, and another from Vicien and Rubinstein is about the successful implementation of a graduate certificate on risk analysis for the Agrifood sector at the University of Buenos Aires.

Finally, an opinion from Gray et al. is included in this Research Topic to honour the memory of a treasured member of the ISBR community, the late Professor Alan Raybould. ISBR gratefully acknowledges the contribution from all the authors to this Research Topic. The society has identified an important niche to fill in the scientific community, and the diversity of topics and article types published as part of this Research Topic exemplify the goals and impact of the ISBR Symposium. The society intends to continue to bring together this unique group to share perspectives, learn from experiences and plan for sound scientific global approaches to biosafety in the future. The 17th ISBR Symposium will take place in November 2025 in Ghent, Belgium.

